# Semantically Guided Large Deformation Estimation with Deep Networks

**DOI:** 10.3390/s20051392

**Published:** 2020-03-04

**Authors:** In Young Ha, Matthias Wilms, Mattias Heinrich

**Affiliations:** 1 Institute of medical informatics, University of Luebeck, 23558 Luebeck, Germany; heinrich@imi.uni-luebeck.de; 2 Department of Radiology, University of Calgary, Calgary, AB T2N 4N1, Canada; matthias.wilms@ucalgary.ca

**Keywords:** image registration, large deformation, weakly supervised

## Abstract

Deformable image registration is still a challenge when the considered images have strong variations in appearance and large initial misalignment. A huge performance gap currently remains for fast-moving regions in videos or strong deformations of natural objects. We present a new semantically guided and two-step deep deformation network that is particularly well suited for the estimation of large deformations. We combine a U-Net architecture that is weakly supervised with segmentation information to extract semantically meaningful features with multiple stages of nonrigid spatial transformer networks parameterized with low-dimensional B-spline deformations. Combining alignment loss and semantic loss functions together with a regularization penalty to obtain smooth and plausible deformations, we achieve superior results in terms of alignment quality compared to previous approaches that have only considered a label-driven alignment loss. Our network model advances the state of the art for inter-subject face part alignment and motion tracking in medical cardiac magnetic resonance imaging (MRI) sequences in comparison to the FlowNet and Label-Reg, two recent deep-learning registration frameworks. The models are compact, very fast in inference, and demonstrate clear potential for a variety of challenging tracking and/or alignment tasks in computer vision and medical image analysis.

## 1. Introduction

Estimation of motion and deformation between images continues to play an important role for multiple vision tasks. While the computation of optical flow for 3D scene motion of smaller magnitude can be performed very effectively and accurately using a range of variational (coarse-to-fine) as well as deep-learning models (e.g., FlowNet [1]), larger deformations still pose a significant challenge. We hypothesize that the large performance gap is due to two reasons. First, current approaches have a limited capture range, in particular for low-textured regions and remain prone to local optima despite extensive multi-scale processing. Second, even supervised approaches that are trained on huge datasets with ground truth flow fields fail to learn sufficient scene understanding and tend to rely on low-level visual clues that become ambiguous for large deformations. Furthermore, learning primarily from synthetically generated ground truth flow fields limits the practical application of deep-learning optical flow models, because adaptation to a new unseen domain is usually not possible and supervised dense correspondences hard to define for real world images.

Weakly supervised approaches [2,3], in contrast, only require semantic segmentation labels to define a loss directly based on the warped segmentation masks for the training of a convolutional neural network (CNN)-based registration model. Yet, these previous works have not addressed large deformation problems and the learned features may again be prone to ambiguous matches (even in combination with smoothness regularization). We therefore propose to use semantic guidance information directly into the feature learning of a two-step deep spatial transformer network for face alignment. Our model comprises a U-Net part that extracts semantic object information for both considered images. This information is fed into flow predictors that rely on a B-spline parameterization for smooth deformable transformations. By employing more than one spatial transformer module within a multi-iteration warping framework [1], we can also avoid the use of memory-intensive cost volume approaches [4] that are hard to extend to high-resolution images or 3D problems.

### 1.1. Contributions

Our work is most closely related to the work of Qin et al. [2] that also proposed to learn segmentation and registration jointly, but was limited to a single (recurrent) transformation network and uses shared weights for both segmentation and registration networks. In contrast to the FlowNet [1,5] or the SVF-Net [6] no (pseudo) ground truth deformation fields are required for training our network, but a loss based on the alignment of labels is used instead. Hu et al. [3] also considered this weakly supervised learning based on segmentation information, but did not include this guidance in the feature extraction U-Net, which is crucial for large deformations as our experiments show. (Example code available at: https://github.com/multimodallearning/semantically-guided).

### 1.2. Related Works

Dataset with ground truth deformation fields are difficult to find. Most supervised methods use automatically generated ground truth deformation fields or estimate parameters for deformable transformations [6,7,8] to train the network. They optimize the mean residual distance between ground truth and estimated deformation fields. In the method of Krebs et al. [7], U-Net-like architecture is used to estimate a dense deformation field from the concatenated fixed and moving images, while Sokooti et al. [8] gave image patches as inputs to a Siamese network to estimate a patchwise 3D deformation field. The obvious drawbacks of these methods are that the estimated deformation fields can only be as good as the automatically generated deformation fields and that the trustworthiness of the generated ground truth field cannot be guaranteed.

Some methods deal with this problem by training the network indirectly using available annotation data, such as segmentation or in an unsupervised manner (only based on a predefined similarity metric) using a spatial transformer network to warp the segmentation of the input image [3,9,10,11,12]. De Vos et al. [9] compare the difference between warped moving image and fixed image directly as loss, where a spatial transformer was employed for image warping. In addition to the image intensity difference, the Label-Reg approach of [3] warps the segmentation using spatial transformer and minimizes the difference between the warped moving and fixed segmentation. Various segmentations or labels are given as input to the network and the soft probabilistic Dice is computed as loss at different image scales. In VoxelMorph [12], in addition to unsupervised losses segmentation can also be used as an auxiliary information to improve the accuracy. The use of segmentation for the estimation of the deformation field can be beneficial especially to counter ambiguous correspondences of object parts with locally similar appearance.

Within computer vision, there are several methods which jointly estimate semantic segmentation and optical flow for videos [13,14,15,16]. Recent works integrate learning into optical flow frameworks to improve the flow estimation using semantic segmentation. Sevilla et al. [13] use localized layers to deal with the movement of different objects in videos. Cheng et al. [15] combine two existing network architectures with a small modification into a network with two branches. Feature maps generated by each branch (one for segmentation, the other for optical flow) are propagated to the other branch to fuse the information for a final output. Hur et al. [14] employ superpixels to connect optical flow and semantic segmentation. Tsai et al. [16] use an iterative scheme to jointly optimize optical flow and segmentation to obtain video segmentation with improved object boundaries. In the medical image domain, Qin et al. [2] have adopted the joint estimation of segmentation and motion for cardiac MR image sequences. For both segmentation and motion estimation, the same network, i.e., with shared weights, is used for feature extraction.

## 2. Materials and Methods

**Figure 1:**
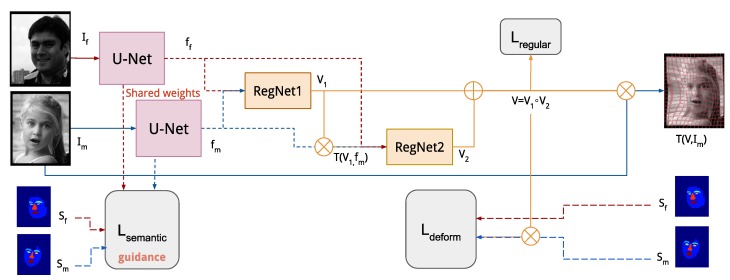
Overview of our framework. From input images $I_{f},I_{m}$ the semantic features $f_{f},f_{m}$ are extracted by U-Net and passed on to the registration networks (RegNet) after the concatenation. Deformation field $V_{1},V_{2}$ are then estimated by the first and the second RegNet, respectively. As input of the second RegNet, the semantic features of the moving image are transformed using the output of the first RegNet. Finally, two deformation fields are combined to obtain the final deformation field V.

A general overview of our proposed method is shown in Figure 1. Our deep network architecture consists of three parts. The first part aims to extract semantically meaningful information using a U-Net structured network [17] and is supervised using a soft constraint on pixel-wise class probabilities using multi-label segmentation. The estimation of the deformation field is carried out in two consecutive convolutional networks with nonlinear spatial transformer modules. Similar to the method of Hu et al. [3], the transformation is only weakly supervised based on the alignment of the warped moving segmentation in comparison with the fixed segmentation. To reduce the number of predicted parameters and ensure a spatially smooth transformation, we use a coarse cubic B-spline transformation model [18] with an additional regularization term.

### 2.1. Semantic Feature Extraction

**Figure 2:**
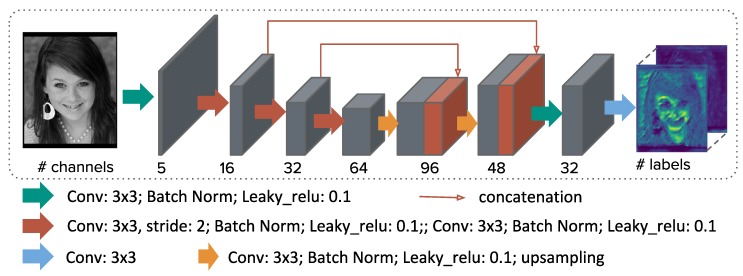
U-Net architecture to extract semantic image features for the subsequent spatial transformer registration network.

To cope with large deformation, we strongly believe that semantic scene information is of great importance. While previous methods [3,6] have implicitly used segmentation information to derive a loss with respect to the estimated transform, we make additional explicit use of semantic clues within the feature extraction for each image individually. Figure 2 shows the U-Net architecture of the feature extraction part of our method, which contains 11 convolution filters with 3×3 kernels, two skip connections and roughly 200k trainable parameters. Given a greyscale image $I\in R^{H\times W}$, the network produces a SoftMax prediction $f\in {\left[0,1\right]}^{N}$ for each pixel and each label l∈1,…L. The semantic loss is computed using weighted cross-entropy:

(1) $$L_{\mathrm{semantic}}=-\sum_{j}^{N}w\hat{f}\left(x_{j}\right)\log f\left(x_{j}\right),$$

where $\hat{f}$ is the ground truth segmentation with *N* sample points and $x_{j}$ is the *j*-th sample point. The label weights vector $w\in R^{L}$ is determined by computing the square root of the inverse class frequency for each label. For our task of registering two input images $I_{f}$ and $I_{m}$, the network weights are shared.

### 2.2. Registration Network

Estimation of the deformation field is performed in two steps, given the network outputs $f_{f}$ and $f_{m}$ of the feature extraction network. The extracted features contain semantic information of the input images, which is the class probability of each pixel. These are passed on to the registration network after concatenation, which is trained to learn a low-parametric deformation field representation $\tilde{V}$ in a smaller dimension. Deformation field $V\in R^{2\times H\times W}$ in the original image size can be retrieved by upsampling using a cubic B-spline function. The evaluation of a dense displacement field using B-splines (also called free form deformations [18]) can be efficiently calculated using three consecutive average pooling layers without stride, following the theory of recursive cardinal splines.

In our two-step setting, we obtain two different deformation fields $V_{1}$ and $V_{2}$ from the first and second registration network, respectively. Both networks have the same architecture; however, the first network is trained with the immediate output of the feature extraction network $f_{f}$ and $f_{m}$, while the second network is trained with the warped moving features $V_{1}\circ f_{m}$ instead of $f_{m}$. The final deformation field V is generated by combining the two fields. In this paper, we combine the fields in two different ways; addition and transformation.

Finally, with the resulting dense displacement field V, the ground truth segmentation of the moving image $S_{m}$ is warped and the deformation loss is computed as follows:

(2) $$L_{deform}=\frac{1}{L}\sum_{l=1}^{L}\;w_{l}\;\left|\;S_{f}\left(l\right)\;-\;T\left(V,S_{m}\left(l\right)\right)\;\right|,$$

where $w_{l}$ denote the label weights and T is the spatial transformer for the B-spline transformation. An additional loss term $L_{\mathrm{regular}}$ for regularization loss is computed for the estimated deformation field, which penalizes deviations between the final estimation of deformation field V and a locally smoothed version of the same displacements field $V_{smooth}$:

(3) $$L_{\mathrm{regular}}=||V-V_{smooth}{\left|\right|}_{2}\;.$$

Finally, overall loss for semantically guided deformation estimation is computed as follows:

(4) $$L=\lambda_{s}\;L_{semantic}+L_{deform}+\lambda_{r}\;L_{regular}\;,$$

where $\lambda_{s}$ and $\lambda_{r}$ are the weight parameters.

### 2.3. Experiments

We have performed ablation studies for our framework and potential variants using the Helen dataset [19]. Our method is also tested on a publicly available medical cardiac dataset (ACDC [20]). The Helen dataset consists of 2330 face RGB images in different image sizes as well as ten segmentation labels for face, eyes, eyebrows, nose, mouth and hair provided by Smith et al. [21]. The ACDC dataset consists of 100 images of 4D MRI for training, for which three segmentation labels of right and left ventricle and myocardium are given.

#### 2.3.1. Data Preprocessing

For the face dataset, we cropped the images to have the same image size of 320×260 using an enlarged face bounding box and converted them into the greyscale images. For training, 2000 images were used as in the work of Le et al. [19] and for the test, the remaining 330 images are used. For our experiment only 7 labels are used, which excludes the hair label, due to their obscure appearance and the mouth structures (upper lip, lower lip and inner mouth) are combined as one single mouth label, since the inner mouth is not given in many samples.

The medical cardiac MRI dataset consists of 100 training image pairs (end-diastolic and end-systolic) with the ground truth segmentation, which we divide into two subsets with 70 and 30 image pairs for training and testing respectively. The original images have a pixel spacing of 1.56 mm for in-plain slice and 5–10 mm of slice thickness. We resampled and cropped all images to have the same voxel dimension 128×128×64 with the slice thickness of ≈1.56 mm.

#### 2.3.2. Implementation Details

The U-Net architecture used to extract semantic features is outlined in Section 2.1 and visualized in Figure 2. To account for small structures in the images, a weighted cross-entropy loss was used. Eleven 3 × 3 convolutional layers and two skip connections with a relatively small total number of 200’000 network parameters were used to avoid overfitting. Downsampling of the input is done using a stride size of 2 in three convolutional layers and two upsampling layers are used to obtain output. The output has half the input size. The network receives an image as input and returns SoftMax probabilities for each structure as a channel.

**Figure 3:**
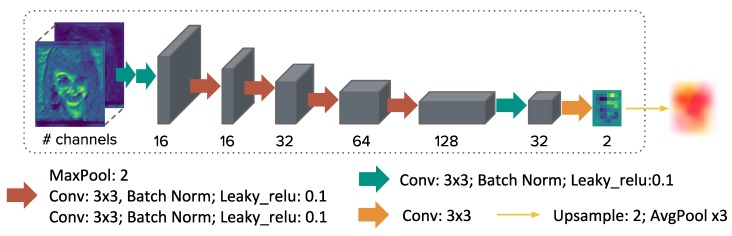
Architecture of our registration network, which estimates displacements of a nonlinear B-spline grid for spatial transformations.

For the registration part, a convolutional network was implemented as shown in Figure 3. As described in Section 2.2, the same architecture is used twice to build a two-step framework with substantially more channels to accommodate the challenging matching problem and optimize both spatial transformations simultaneously during training. The output of registration network has a smaller parametrization than the image. Therefore, we upsample the control-point displacement and apply three cardinal B-spline smoothing steps before warping using average pooling layers. The kernel size of the average pooling layers and thus the scale of the B-spline transformation for the output is chosen to be 19 for the first network part (to capture large and coarse deformations) and 11 for output of the second network (for a refinement of small structure alignment). For the medical dataset, the scales of 5 and 3 are used, because the motion was smaller on average for this dataset. The scale was chosen to be approximately 5% and 3% respectively (for first and second registration network output) of the largest image dimension for the B-spline transformation, based on empirical tests. The smoothing kernel size was also set relative to image size and object size (in particular the following values were chosen: 5 and 3 for the face and the medical dataset respectively).

Throughout our experiment, we use Adam optimizer with the learning rate of 0.001 and the momentum of 0.97. The weight of the regularization loss $\lambda_{r}$ was chosen to be 0.001 and the weight of semantic loss $\lambda_{s}$ 1.0, which were determined empirically. For the regularization, the estimated dense field is smoothed using two average pooling layers with the kernel size of 3 for the medical dataset and 5 for the face dataset, also chosen empirically. The training batch size for the face dataset was 20 and for the medical dataset 5. The training was performed for 300 epochs for both datasets.

#### 2.3.3. Evaluation Metric

Given a deformation field for a pair of test images, we use the mean of the Dice coefficient (equivalent to F1 score) across individual face parts as well as the contour distance of each structure to evaluate the alignment (or registration) quality. Dice score for a label is computed as:

(5) $$Dice=\frac{2\sum_{i}\left|a_{i}b_{i}\right|}{\sum_{i}|a_{i}|+\sum_{i}\left|b_{i}\right|}$$

where $a_{i},b_{i}$ are the labels in pixel *i* of reference image and target image, respectively. Contour distance of each structure is computed as:

(6) $$D_{contour}=\frac{\sum_{s_{a}\in S\left(A\right)}d\left(s_{a},S\left(B\right)\right)+\sum_{s_{b}\in S\left(B\right)}d\left(s_{b},S\left(A\right)\right)}{\left|S(A)|+|S(B)\right|}$$

where S(A) and S(B) are the total contour pixels of the reference and target images, respectively. The mean Dice values are computed between the ground truth segmentation of fixed images and warped ground truth segmentation of moving images. Please note that the segmentation labels for guidance are only available for training images and new test pairs are registered without any manual labels. The labels are only used to evaluate the registration accuracy. In addition to the alignment accuracy, we have evaluated the quality of the estimated transformations, which is crucial for subsequent vision applications such as style transfer. We calculate the Jacobian determinant (also *Jacobian*) to provide a quantitative evaluation on the topology of the deformation field.

For each pixel (*x*,*y*), *D*(*x*,*y*) is the estimated displacement vector.

(7) $$Jacobian=det\left[I+\left(\begin{array}{cc} \frac{\delta D_{x}\left(x,y\right)}{\delta x} & \frac{\delta D_{x}\left(x,y\right)}{\delta y}\\ \frac{\delta D_{y}\left(x,y\right)}{\delta x} & \frac{\delta D_{y}\left(x,y\right)}{\delta y} \end{array}\right)\right]$$

where *I* is the identity matrix, $D_{x}\left(x,y\right)$ and $D_{y}\left(x,y\right)$ are the x and y component of D(x,y) respectively. A Jacobian in each pixel provides the characteristic of the deformation, i.e., a Jacobian of 1 for no change, >1 indicate expansion, 0–1 shrinkage and a Jacobian ≤0 indicates singularity. We report standard deviation of the Jacobian, where smaller values indicate smoother transforms. The number of negative Jacobian determinants indicates the number of singularity points, i.e., with smaller mean number of pixels with a negative Jacobian, the deformation is more plausible.

In the following, different settings are compared to analyze the effect and importance of each setting.

## 3. Results and Discussion

### 3.1. Ablation Study

In this section, different parts of our framework are evaluated and compared to determine the importance of each part. Our experiments compare minor modifications to the training scheme of our proposed method: with or without guidance, with or without shared weights in the feature extraction, and diffeomorphic transformation models.

#### 3.1.1. Single vs. Two-Step Registration

**Figure 4:**
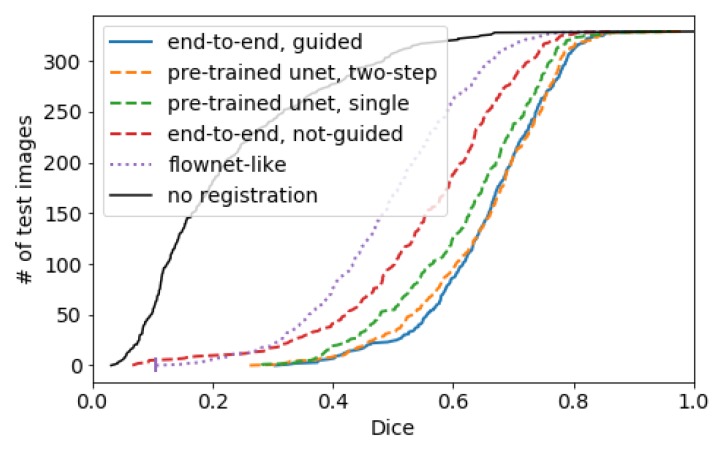
Sorted Dice scores (averaged across face structures) for all test images for various network configurations (lower line is better). Details in Section 3.1.

In many cases, the estimation of deformation field is performed in a single step. We believe that dividing the registration process into smaller steps by using two networks with the same total number of parameters is more beneficial than estimating deformation in a single step. In the two-step registration, one of the input features is transformed with the output deformation field from the first registration network before being forwarded to the second one. We compare the performance of the two-step network with the performance of the single network, where both networks have a total of approximately 2.3 million network parameters. As shown in Figure 4, the two-step network (pre-trained unet, two-step) outperforms the single network (pre-trained unet, single).

#### 3.1.2. Regularization

**Figure 5:**
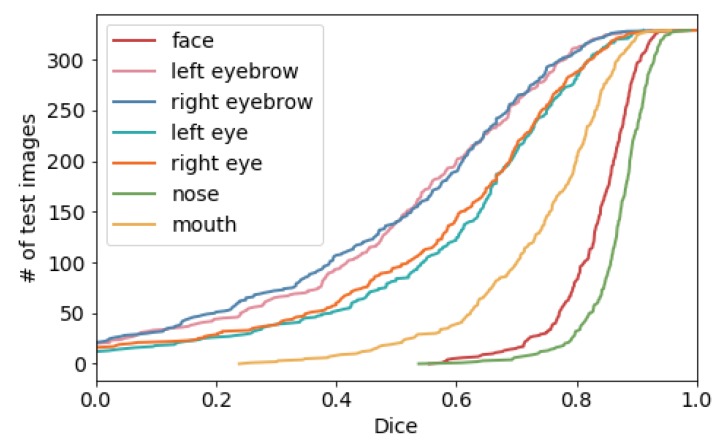
Sorted Dice scores for different face structures. The alignment of eyes and eyebrows is challenging due to occlusions caused by hair in many images.

As described in Equation (3), our regularization term penalizes the difference between non-smoothed and smoothed output fields and is scaled by $\lambda_{r}$. Without the regularization loss the networks could not learn to estimate any plausible deformation field. We use average pooling layers for computation of smoothed deformation fields, where the kernel sizes of the pooling layers are chosen to reduce the standard deviation of the Jacobian of deformation fields. Using a too large kernel size might deteriorate the accuracy of the deformation fields with respect to smaller structures. Dice values for each structure are shown in Figure 5 for the empirically chosen kernel size of 5. Although we found this size to be usually most appropriate, for small structures such as eyebrows and eyes approximately 10% are not registered at all. However, this might also have been influenced by the occlusion due to hair, especially when the forehead is covered.

#### 3.1.3. Semantically Guided Deformation Estimation

**Figure 6:**
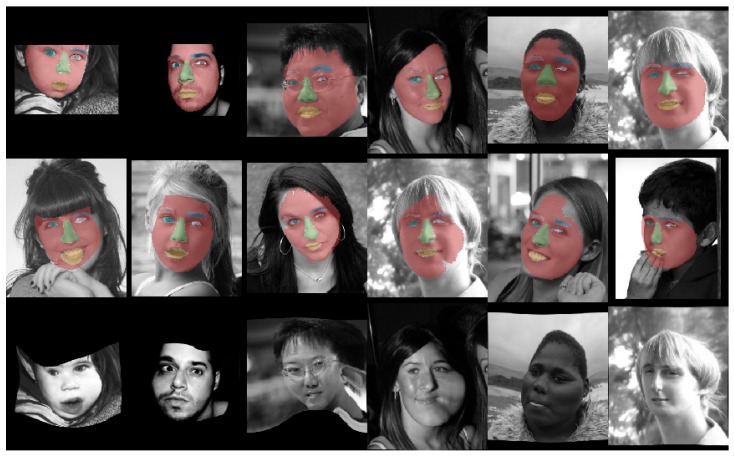
Example results of our approach. (Top) fixed image with the ground truth fixed labels, (middle) target image with warped ground truth fixed labels, (bottom) warped fixed images.

**Figure 7:**
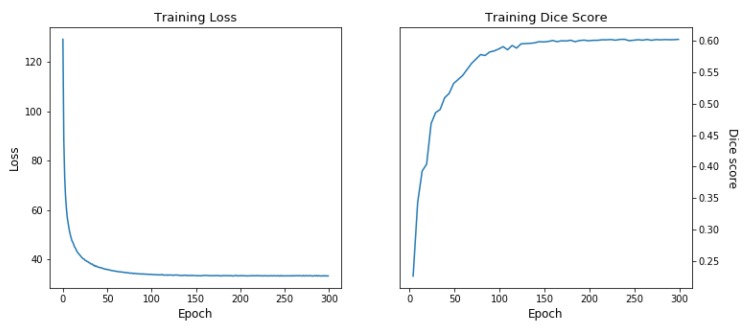
Training loss and accuracy curves of the guided network (two-step, end-to-end). The training loss and accuracy graph of other variations of our experiment show similar curves and therefore left out.

Instead of using a pre-trained segmentation network to start the deformation estimation, we jointly train the semantic feature extraction U-Net with the two-step networks using semantic loss (see Figure 4: end-to-end, guided and Table 1: ours) and compare with the same configuration without semantic loss using only the deformation and regularization loss (see Figure 4: end-to-end, not-guided and Table 1: ours (without guidance)). This has the advantage that only a soft constraint is used for the semantic guidance loss and the deformation network can use clues based on more generic image features (e.g., edges). However, this only led to a small quantitative improvement of the alignment in terms of Dice score (0.5%), the warped images appear to be more realistically transformed. Example results of our method is shown in Figure 6 and the training curves are shown in Figure 7.

#### 3.1.4. Diffeomorphic Transformation

The final deformation fields from our method are smooth and the percentage of pixels with negative Jacobian is relatively small (≈0.1%), albeit the fields are not diffeomorphic. We have compared two cases where diffeomorphic deformation fields are computed. First, we estimate stationary velocity fields instead of deformation fields and compute the final field using scaling and squaring to obtain diffeomorphic transformation (see Table 1: ours (diffeomorphic)). Second, we apply a poly-affine transformation as post-processing step to reduce the singularities in the resulting field (see Table 1: ours (poly-affine)). The result shows that with the decreased singularity in the resulting field, the accuracy of the transformation is also reduced. The singularity present in the final fields could be necessary, e.g., to compensate for occlusion in the images.

### 3.2. Comparison with Other Methods

The registration results with different quantitative evaluation measures are shown in Table 1. As long as it was possible, we have evaluated the methods using Dice score, contour distance, standard deviation of Jacobian and the mean number of pixels with negative Jacobian.

We compare our method with various methods; however for this experiment we have implemented our own version of the networks, where we use same loss function as in the original methods except for FlowNet. We use the original FlowNet version and used the pre-trained model. Solely for this case, we have downsampled the input images and performed an affine transformation beforehand (which constitutes an easier registration task). For the reproduction of Qin et al.’s method [2], we used shared weights for registration networks, which are however, not shared with the segmentation network. The reason for this was to compare the effect of using a two-step registration network against recursive training of registration networks.

A new variant that combines the correlation layer of the FlowNet with our proposed B-spline parameterization was implemented. This B-Spline FlowNet (Table 1: B-Spline FlowNet) yields significant improvements compared to the original FlowNet (Table 1: FlowNet w/ smaller images) but less accurate alignment (more than 10% points gap) than our proposed method. This clearly demonstrates that large deformations are very hard to estimate without meaningful semantic guidance during the training process.

We also compare our networks that are learned with segmentation information with strongly supervised landmark models. As an example approach of this category, we used explicit face regression of Cao et al. [22] to predict landmarks for all test images. Subsequently, we calculated a dense warp field using the coherent point drift algorithm [23] (Table 1: ESR + CPD). Please note that corresponding landmarks are much harder to annotate than segmentation labels and in many applications, in particular 3D medical images, no meaningful points can be selected at all. This means that landmark-model-based registration is somewhat out of competition and could be seen as an upper bound to employing supervision with segmentations only. Nevertheless, our approach achieves comparably high Dice overlap scores to the method of Cao et al. [22] with only slightly more complex transformations (reflected by higher Jacobi determinants).

### 3.3. Medical Cardiac Image Registration

**Figure 8:**
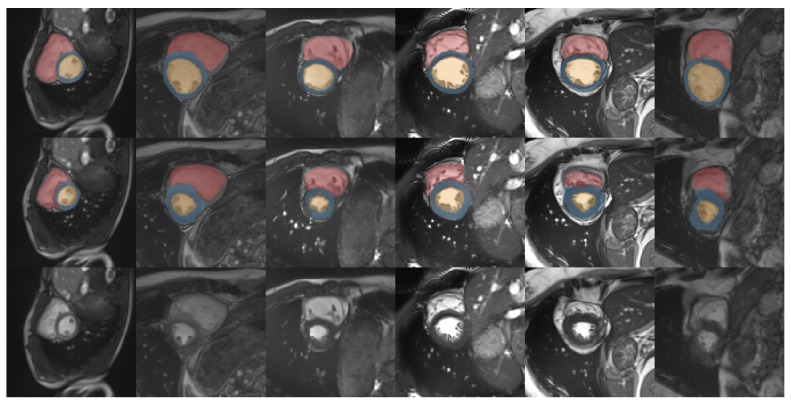
Example results (example slices) of our approach for intra-patient registration. (**Top**) end-systolic slices with the corresponding ground truth labels, (**middle**) end-diastolic slices with warped ground truth labels of end-systolic slices, (**bottom**) warped end-systolic slices.

We evaluate our semantically guided registration network for the preprocessed MRI images from the ACDC challenge [20]. The difficulty here lies in the large motion and strong imaging artefacts. In addition, the substantial differences in appearance and contrast across subjects make learning a model that generalizes well for a population difficult. Therefore, many previous approaches have employed unsupervised learning strategies [24,25] or classical optimization-based methods [26], which can rely on comparable intensity levels within one patient. We compare the results of these three methods (Dice scores of compared methods are reported by Krebs et al. [25]) with our semantically guided framework. As shown in Table 2, our method outperforms all these state-of-the-art methods for the alignment of all structure. The resulting deformations are also plausible with a standard deviation of Jacobian determinant of 0.3. Example results of intra-patient image registration are shown in Figure 8.

## 4. Conclusions

We have presented a new semantically guided and two-stage deep deformation network that can be trained end-to-end and excels at registering image pairs with large initial misalignments. Our extensive experimental validation shows that employing semantic labels available only during training for both an alignment loss and a soft constraint on correct segmentation prediction yields superior results compared to previous approaches that have only considered the former one. Moreover, the use of two-stage networks improves the accuracy compared to a single network or two networks with shared weights. This guidance can also be used beneficially in a series of multiple spatial transformers to improve the alignment of particularly challenging image pairs. Our results on both the Helen face dataset and the medical cardiac ACDC data improve upon the state of the art including FlowNet [1] and Label-Reg [3]—two very recent deep-learning registration frameworks—as well as several unsupervised approaches. Our resulting models are compact and very fast in inference (≈0.009 s per image pair) and can be employed for a variety of challenging tracking and/or alignment tasks in computer vision and medical image analysis. 

## Figures and Tables

**Table 1:** Dice scores, contour distances in pixel and the standard deviation of Jacobian determinant of estimated deformation fields using different methods (for the Helen dataset). The standard deviation of Jacobian determinants of deformation fields show the smoothness of the deformation fields, where small values indicate more plausible, regular results. The mean of Jacobian negatives is the number of singular points in the estimated field divided by the total number of pixels. For B-Spline FlowNet, ours (without guidance) and ours (shared RegNet weights), we have implemented our own version of the method from the each reference (details in Section 3.2). Explicit shape regression (ESR) requires in addition corresponding manual landmarks for training. The experiment using pre-trained FlowNet was performed without fine-tuning and using downsampled images, which we first affine transformed based on the face landmarks. Approximated inference time is also given in seconds per image.

Method	Dice (%)	Contour Distance (px)	Jacobian Std.	Jacobian Negatives	Inference Time (s/img)
no registration	23.0	15.55	-	-	-
ESR ([22]) + CPD ([23])	65.6	**1.96**	0.154	-	-
B-Spline FlowNet	49.4	5.96	0.579	0.03124	≈0.007
FlowNet w/ smaller images ([1])	30.6	12.83	-	-	-
ours (without guidance, ∼[3])	55.5	5.41	0.257	0.00062	≈0.007
ours (shared RegNet weights, ∼[2])	60.4	5.02	0.269	0.00061	≈0.007
ours (diffeomorphic)	52.0	5.66	0.240	0.00062	≈0.007
ours (poly-affine)	65.8	3.95	0.281	0.00093	≈0.024
**ours**	**66.0**	4.01	0.285	0.00106	≈0.007

**Table 2:** Mean Dice scores (%) of different methods for medical cardiac dataset averaged across 30 test subjects by labelled structures (R.V.: right ventricle, L.V.: left ventricle).

	Unregistered	LCC-Demons [26]	Voxel-Morph [24]	Krebs et al. [25]	Ours
R.V.	65.1	70.6	68.1	68.4	**77.4**
L.V.	66.0	77.6	74.3	75.6	**82.5**
Myocard	52.5	73.0	69.7	70.4	**73.4**
**Mean**	61.2	73.7	70.7	71.5	**77.8**
